# The effects of the presence of a face and direct eye gaze on voice identity learning

**DOI:** 10.1111/bjop.12633

**Published:** 2023-01-23

**Authors:** Nadine Lavan, Nisha Ramanik Bamaniya, Moha‐Maryam Muse, Raffaella Lucy Monica Price, Isabelle Mareschal

**Affiliations:** ^1^ Department of Biological and Experimental Psychology, School of Biological and Behavioural Sciences Queen Mary University of London London UK

**Keywords:** automatic attention capture, direct gaze, face overshadowing effect, voice identity

## Abstract

We rarely become familiar with the voice of another person in isolation but usually also have access to visual identity information, thus learning to recognize their voice and face in parallel. There are conflicting findings as to whether learning to recognize voices in audiovisual vs audio‐only settings is advantageous or detrimental to learning. One prominent finding shows that the presence of a face overshadows the voice, hindering voice identity learning by capturing listeners' attention (Face Overshadowing Effect; FOE). In the current study, we tested the proposal that the effect of audiovisual training on voice identity learning is driven by attentional processes. Participants learned to recognize voices through either audio‐only training (Audio‐Only) or through three versions of audiovisual training, where a face was presented alongside the voices. During audiovisual training, the faces were either looking at the camera (Direct Gaze), were looking to the side (Averted Gaze) or had closed eyes (No Gaze). We found a graded effect of gaze on voice identity learning: Voice identity recognition was most accurate after audio‐only training and least accurate after audiovisual training including direct gaze, constituting a FOE. While effect sizes were overall small, the magnitude of FOE was halved for the Averted and No Gaze conditions. With direct gaze being associated with increased attention capture compared to averted or no gaze, the current findings suggest that incidental attention capture at least partially underpins the FOE. We discuss these findings in light of visual dominance effects and the relative informativeness of faces vs voices for identity perception.

## BACKGROUND

When becoming familiar with the voice of a person, we rarely learn to recognize their voice in isolation. Instead, we often become familiar with a person in multimodal settings, such that we learn to recognize the person's face (and any number of other aspects of this person) at the same time as we learn to recognize their voice. What is the effect of having access to additional facial information while learning to recognize a new voice? Overall, faces tend to be the more reliable cue to person identity and accuracy of face identity perception often better than accuracy of voice identity perception (Barsics, 2014; Young et al., 2020; but see Kanber et al., 2022). Therefore, faces could form a valuable anchor point during voice identity learning, potentially offering access to partially shared identity‐related information and might thus support voice identity learning. Conversely, faces may interfere with voice identity learning, as listeners may focus on the face as the more reliable signal. Faces may thus draw attention away from the voice and impair voice encoding.

There are conflicting findings in the literature regarding whether the presence of a face helps or hinders voice identity learning. Previous studies have, for example, shown that being presented with audiovisual information, that is, seeing a face while listening to a voice, can enhance voice identity learning (Maguinness et al., 2021; Schall et al., 2013; Sheffert & Olson, 2004; Von Kriegstein et al., 2008; Von Kriegstein & Giraud, 2006; Zäske et al., 2015). In these studies, listeners typically learn to recognize a number of voice identities during training tasks. During training, listeners are either presented with dynamic audiovisual stimuli, including the face and voice of the to‐be‐learned identities, with the voices only (Sheffert & Olson, 2004; Zäske et al., 2015) or with cues to another aspect of their personal information, such as the person's occupation (e.g. Maguinness et al., 2021; Schall et al., 2013; Von Kriegstein et al., 2008; Von Kriegstein & Giraud, 2006). Across the studies, accuracy for voice recognition after training is reported to be higher following the audiovisual training, i.e., when a face was present, compared to audio‐only training.

With participants being able to form an audiovisual representation of a person during such audiovisual training, it has been proposed that the additional identity‐related information the faces provide can be used during audio‐only voice identity recognition. Specifically, when being only presented with the voices (but not the faces) listeners are thought to nonetheless simulate the static facial features during such audio‐only voice identity recognition. That is listeners may automatically call to mind the face when hearing a voice and use information encoded in the face of a person about their identity to support accurate voice recognition (e.g. Maguinness et al., 2021; Von Kriegstein et al., 2008). This account of the “face benefit” during voice identity learning is based on a mechanistic account of how audiovisual training can enhance audio‐only speech perception with participants having access to additional information about the speech encoded in the articulatory movements visible in the face. For identity perception, this account thus strongly assumes that there is meaningful shared and complementary identity‐related information across the auditory and visual modalities. While there are studies that suggest that there is limited shared information, others, however, report low or close to chance‐level accuracy for any cross‐modal identity matching (see Lavan, Smith, et al., 2021; Smith et al., 2016a, 2016b).

In contrast to these reports of face benefits during audiovisual voice identity training, there is also a literature reporting the opposite effect. Focusing mainly on voice identity perception in forensic contexts, these studies find that audiovisual training can in fact be detrimental to voice identity learning across varied experimental tasks. For example, Cook and Wilding (1997, 2001) presented listeners with a recording of two target voice identities saying one sentence each. The voices were either presented on their own without any visual information or were presented as part of a video that also showed the faces of these two target identities. One week after this very brief exposure phase, participants were then presented with audio‐only voice line‐ups, including the two target voices plus a number of foil voices. Listeners were more accurate at identifying the target voices from the voice line‐ups following audio‐only exposure than when they had learned the voices through audiovisual exposure. Similarly, Stevenage et al. (2011) and Tomlin et al. (2017) asked listeners to complete a short exposure phase during which they rated the to‐be‐learned voices for attractiveness. This was followed by an “old”/“new” voice identification task including the previously learned “old” voices and a number of previously unheard “new” voices. These two studies also report worse performance for audiovisual training compared to audio‐only training. This disadvantage or “Face Overshadowing Effect” has primarily been interpreted as an attentional effect. Since the face is a more reliable signal to identity, it has been proposed that the presence of a face automatically captures participants' attention and interferes with the encoding of the voice identities (Cook & Wilding, 1997, 2001; Stevenage et al., 2011; Tomlin et al., 2017).

Given these conflicting findings regarding the effects of audiovisual training on voice identity learning, it is unclear what drives the differences in reported effects across studies. Some efforts have been made to reconcile the differences as simply being results of experimental design choices, although this led to further somewhat conflicting findings. For example, it has been reported that the amount of exposure time to individual voices during the training may affect the direction of the reported effect. Zäske et al. (2015) report *benefits* of audiovisual training are only apparent after a moderate amount of training per voice (~ 6 s of read speech * 5 to 6 repetitions), while *disadvantages* were present for very brief amounts of training (~6 s of read speech * 1 to 2 repetitions). Zäske et al. (2015) suggest that these findings reflect that faces initially capture participants' attention, thus hindering voice learning. With longer training, participants may, however, re‐allocate their attention to the voice, alongside the face, yielding face benefits. Cook and Wilding (2001) also suggest that longer exposure diminishes the Face Overshadowing Effect, although this difference between conditions in their study is not statistically significant. Finally, Legge et al. (1984) describe effects of exposure time, although unlike in the previous studies, they find no clear linear trends that would suggest that longer exposures are associated with face benefits and no formal statistical analysis is reported.

In addition to effects of the duration of exposure or training, studies have also explored whether differences in stimulus properties may affect how successful voice identity learning is during audiovisual training. Zäske et al. (2015) examined the effects of static vs dynamic face stimuli, reporting that effects of audiovisual training can be observed when using both dynamic and static visual stimuli. For static faces, there are greater disadvantages after audiovisual training with shorter exposure for voice identity learning and smaller advantages for longer exposure per voice. The opposite was true for dynamic faces. In contrast, however, Tomlin et al. (2017) report no difference in voice identity recognition after static and dynamic audiovisual training. Similarly, Tomlin et al. (2017) report that the Face Overshadowing Effect disappears entirely if inverted faces are presented during training, suggesting that this effect is face‐specific and is not driven by low‐level stimulus properties. Overall, there is therefore some evidence that experimental design choices and stimulus properties can modulate the effect of the presence of a face on voice identity learning, although no consistent picture emerges that may point to the cognitive mechanisms underpinning the effects and its modulations.

Beyond trying to explain conflicting findings through examination design or stimulus‐based features of previous experiments, there have been some attempts in the forensic literature to examine the proposed mechanisms underpinning the Face Overshadowing Effect. Cook and Wilding (2001) investigated whether effects of audiovisual training may be linked to attention by manipulating the task instructions, either by asking their participants to specifically pay attention to the voice, or by not guiding participants' attention towards either modality. This manipulation of attenttional focus did not affect the Face Overshadowing Effect, with audiovisual training impairing voice identity recognition equally for both listener groups. The authors therefore argue that the Face Overshadowing Effect may be a result of automatic attention capture by the faces, with listeners being unable to re‐allocate attention to the voices even when directly instructed to do so (see also Stevenage et al., 2011).

In the current study, we aimed to first replicate and further examine whether and how the presence of visual information (i.e. a face) would affect how well listeners learn to recognize the identities of a set of voices. We furthermore investigated how one proposed mechanism – automatic attention capture – may modulate the reported effects of audiovisual training on voice identity learning. For this purpose, we asked listeners to learn to recognize a set of four female voices across two training tasks. Listeners were either presented with (1) the voices on their own (Audio‐Only), (2) the voices accompanied by static faces with their eyes closed (No Gaze), (3) the voices accompanied by static faces looking either to the left or to the right (Averted Gaze), or (4) the voices accompanied by static faces exhibiting direct gaze (Direct Gaze). This manipulation of gaze via a change in gaze direction or by removing gaze information in conditions 2–4 allows us to assess the role of attention on audiovisual identity learning: Since direct eye gaze has often been associated with increased attention capture compared to averted and no gaze (e.g. Framorando et al., 2016; Frischen et al., 2007; Lavan et al., 2022; Mares et al., 2016; Senju & Hasegawa, 2005), there should be most attention capture in the Direct Gaze condition. At the same time, there should be comparatively less attention capture in the No Gaze and Averted Gaze conditions, with there being no attention capture when the face is absent during training in the Audio‐Only condition.

In terms of the accuracy at test, we therefore had the following predictions: Since some studies report that visual information is beneficial, while others report detrimental effects, we did not have a directional hypothesis for the differences in accuracy between audiovisual training and audio‐only training. For the role of automatic attention capture on any of the reported effects, we predicted that direct gaze should enhance any effect of the visual stimuli, such that if the presence of visual information is detrimental we would expect voice identity learning to be further impaired in the presence of direct vs when gaze was averted or was not present. Conversely, if the presence of visual information is advantageous, we would expect that voice identity learning would be better in the presence of direct gaze than no or averted gaze stimuli, since increased incidental attention may overall enhance the formation of a proposed multimodal person representation during training.

## METHODS

### Participants

202 participants were recruited via the online platform Prolific.co. All participants were native speakers of English, who were born and currently residing in the UK. They were aged between 18 and 40 years, had no self‐reported hearing difficulties and had normal or corrected‐to‐normal vision. Furthermore, each participant's approval rate for prior studies on Prolific.co was 90% or higher. Two participants were excluded from this sample as they failed the attention check (see Procedure). Thus, the analysis of data from 200 participants (mean age = 30.35 years, *SD* = 6.00 years, 131 female) is reported below. Participants were paid £2.33 for 20 min of participation. The study was approved by the local ethics committee.

### Materials

Participants were asked to learn a set of four female voices in two brief training tasks. After this training phase, participants were presented again with the set of four voices they had just learned plus four previously unheard voices. Based on these previously learned vs previously unheard voices, participants were asked whether a voice was an “old” voice, that was learned during training, or a “new” voice, that was not part of the training and thus previously unheard.

#### Voice samples

We selected eight female speakers from the LUCID corpus (Baker & Hazan, 2011): Half of these eight voices were used during training and test. The other half was used as previously unheard voices at test only in addition to the four trained voices. The assignment of the four voices used during training was counterbalanced across participants (see Procedure). All speakers were young adults (aged between 18–29 years) and were native speakers of Standard Southern British English.

All voice recordings included speakers reading out a list of short sentences in a neutral tone of voice (e.g. “The young children loved the beach”). The training stimuli comprised 24 recordings per voice. All voice stimuli were root‐mean‐square normed for intensity and the average duration of all training stimuli was 1.67 s (*SD* = 0.30 s) and 1.76 s (*SD* = 0.29 s) for all test stimuli. Test stimuli comprised 12 recordings per voice. None of the stimuli used in training was re‐used at test.

#### Face stimuli

We selected three images of four White young adult females from the Radboud Faces Database (Langner et al., 2010): In these images, the person was forward facing with their face up to their shoulders being visible. For each identity, one of the three selected images showed the person looking directly at the camera (Direct Gaze), and the other two images showed the person in the same position, looking either to the left or to the right (Averted Gaze). Finally, we created stimuli showing these four identities with their eyes closed via the image manipulation program GIMP (No Gaze). Image manipulations were sufficiently realistic, such that participants did not comment on detecting the manipulation in a debrief question (“Did you notice anything unusual about the images of the faces?”).

Both the Averted Gaze and No Gaze conditions were included in this experiment, since there is some evidence averted gaze may under some circumstances capture attention to a similar degree as direct gaze (Framorando et al., 2016) or may indeed be processed preferentially compared to direct gaze (Riechelmann et al., 2021). Thus, while the comparison of averted vs direct gaze has been studied extensively in relation to attention capture, it may not at all times be a suitable comparison to measure attention capture. While studied less frequently, the contrast of no gaze (via closed eyes) vs direct gaze may not only potentially be a more appropriate comparision (Framorando et al., 2016), it can also provide another comparison condition contrasting less vs more attention capture of faces via a gaze manipulation.

#### Vigilance stimuli

Vigilance stimuli included recordings of a male voice saying “Please press “old voice”/“new voice” now” created via a text‐to‐speech synthesizer.

### Procedure

The experiment was hosted on Gorilla.sc (Anwyl‐Irvine et al., 2020). Participants first read an information sheet, fill in the consent form for the study, and completed an audio check to ensure that sound playback was working adequately. Participants were then randomly assigned to one of the four experimental conditions in our experiment: (1) Audio‐Only, where participants (*N* = 50) learned to recognize a set of four voices, (2) Direct Gaze, where participants (*N* = 47) learned to recognize the voices while being simultaneously presented with an image of the face of a gender‐matched identity directly looking at the camera. (3) No Gaze, where participants (*N* = 50) learned to recognize the voices alongside the image of a face with their eyes closed. (4) Averted Gaze, where participants (*N* = 53) learned to recognize the voices alongside the image of a face with gaze averted to the left or right of the camera. For the audiovisual conditions, face‐voice pairs were fixed throughout training, such that each voice was only ever associated with one specific face to form an audiovisual identity pair. Crucially, throughout the training phase and for all conditions, participants were asked to learn the voices, as opposed to being asked to learn to recognize, for example, the faces and the voices. We do not expect this type of instruction to affect the reported results, as previous research shows that explicitly directing participants' attention to remembering the voices does not impact accuracy at test (see Cook & Wilding, 2001). We note that since participants completed the experiment online, using their own headphones and computer screens, sounds will have been presented to all participants at a comfortable but variable level. Similarly, the size of the face presented on the screen will have varied depending on the resolution of the screen the participants were using.

**FIGURE 1:**
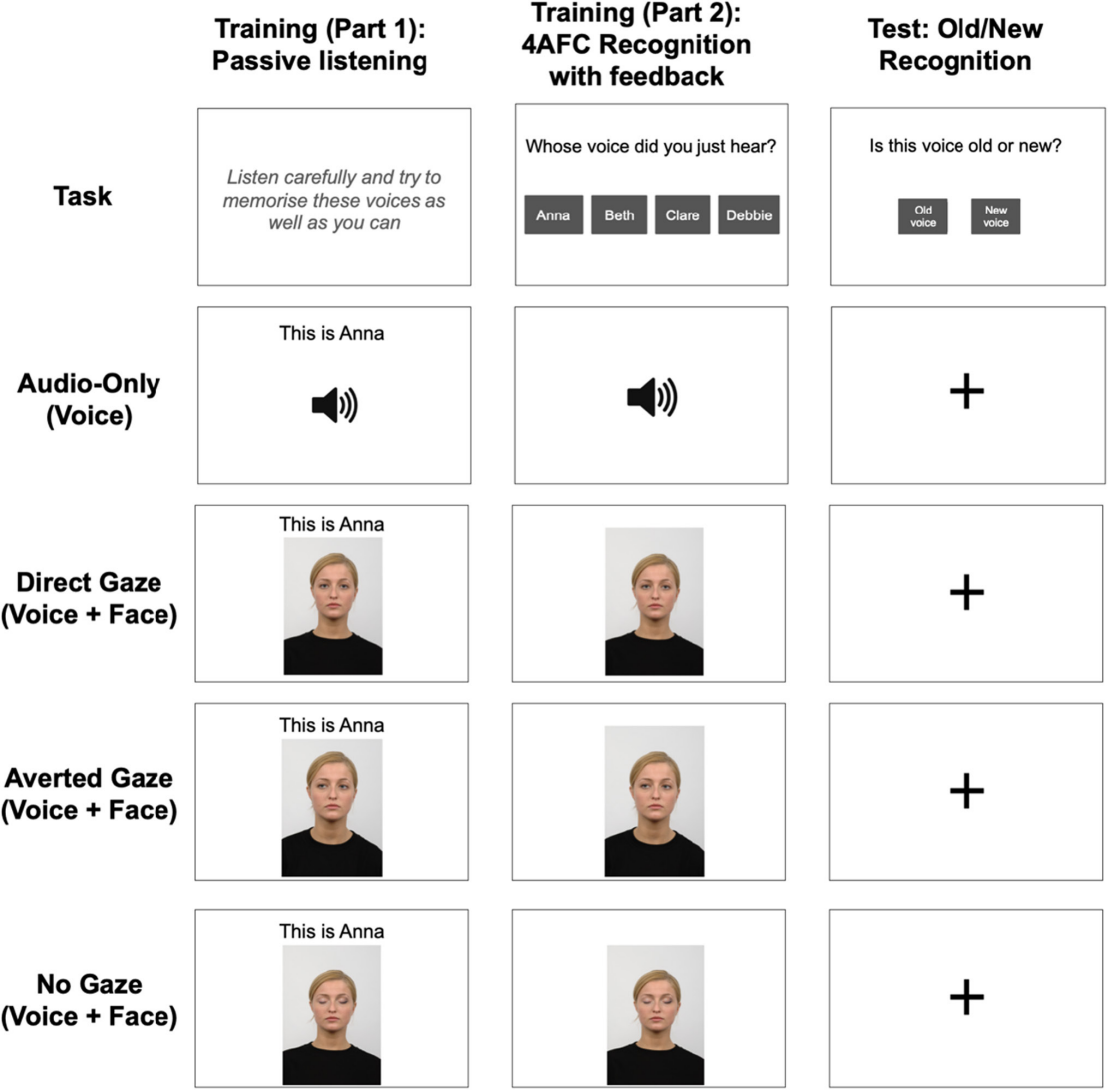
Illustration of the stimuli and experimental design: The first row illustrates the task completed by participants during the two training tasks and at test. The boxes for the four rows below show the visual information presented on the screen while participants were hearing recordings of voices during the two training tasks and at test.

The training phase included two tasks: During the first task, participants were presented with the four voices, where all 24 voice recordings for each identity were presented in blocks, that is, recordings were played one after another, while a text message was presented on the screen, providing participants with the name of the voice (“This is Anna/Beth/Clare/Debbie”, see Figure 1). Participants in all conditions were asked to passively listen to these voice recordings and to try to memorize the four voices and their names. For the Audio‐Only condition, an image of a cartoon loudspeaker was visible on the screen throughout the playback of the recordings to indicate that sound playback was happening. For the Direct Gaze, Averted Gaze and No Gaze conditions, an image of the face that was paired with that voice and name was presented instead of the image of a loudspeaker. In other words, in this first part of the training, participants were presented with a voice and a name in the Audio‐Only condition, while they were presented with a voice, a face and a name in the Direct, Averted and No Gaze conditions. The order in which participants were presented with the different voices was randomized. The direction of the averted gaze was varied, such that for half of the Averted Gaze trials the face was shown looking to the left, while an image of the face looking to the right was shown for the other half of the trials.

After passively listening to all stimuli, participants then completed the second part of the training. In this task, participants were presented with the same set of voice recordings as in the first part of the training but were now asked to identify the voice in each recording by name in a four‐way forced choice task (“Whose voice did you just hear?” Anna/Beth/Clare/Debbie, see Figure 1). Similar to the first part of the training phase, participants saw an icon of a loudspeaker on the screen while hearing the voice recordings for the Audio‐Only condition and were presented with the same pairs of face images and voice recordings they had encountered during the first training task for the Direct, Averted and No Gaze conditions. After selecting a response, participants were then given audiovisual feedback on whether their answer was correct or incorrect, followed in all cases by a screen displaying the name of the correct identity while also replaying the stimuli presented in this particular trial. In total, there were therefore 96 trials (24 recordings * 4 identities) in this second part of the training.

Immediately following this training phase, all participants then completed an old/new recognition task where they heard novel recordings of the four learned voices, interleaved with recordings of four previously unheard voices. For all participants, voice stimuli were presented without any accompanying visual stimulus other than a central fixation cross (see Figure 1). After each voice recording was played in full, participants were asked to indicate whether they thought the voice they just heard was an “old voice” they had learned during training or a “new voice” that they had never heard before (see also Stevenage et al., 2011; Tomlin et al., 2017). Furthermore, vigilance trials were included, during which participants were asked to follow the instructions given to them by the male voice (see Materials). All responses were self‐paced. There were 108 trials in total in this recognition task, comprising 96 experimental trials (12 recordings * 8 voices) and 12 vigilance trials.

After the recognition task, participants finally completed a short debrief questionnaire, where they were able to report any technical difficulties (no significant difficulties were reported for any of the participants). Participants were also asked whether they made a reasonable effort to try to learn the voices and were informed that their answer to this question would not have an impact on their payment. All participants confirmed that this was the case.

All data were analysed in R. Post‐hoc tests were implemented using package *emmeans*.

## RESULTS

### Accuracy during training

We first analysed the mean accuracy during the second training task for each participant. Accuracy was high during training (chance level = 25%) with participants on average identifying the correct identity in a four‐way forced‐choice paradigm in 82.6% of trials in the Audio‐Only condition, 91.4% of trials in the No Gaze condition, 91.9% of trials in the Averted Gaze condition, and 92.4% of trials in the Direct Gaze condition. One‐sample t‐tests indicated that accuracy was well above chance level for all conditions (ts > 34.46, *p* < .001, Cohen's *d* > 2.76).

**FIGURE 2:**
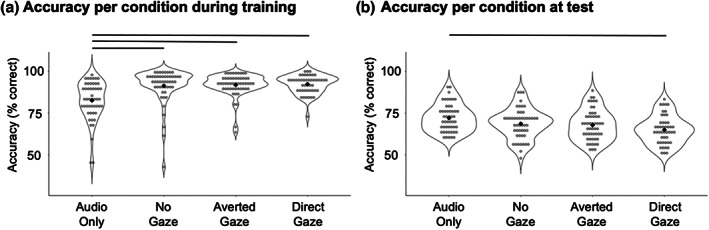
(a) Mean accuracy per participant plotted for each condition during the second part of the training. (b) Mean accuracy per participant plotted by condition at test. Grey dots show means for individual participants. Black *diamonds show th*e *group* means. Lines show significant pairwise comparisons after Bonferroni–Holm correction for six comparisons.

We then conducted a between‐subjects one‐way ANOVA to investigate whether accuracy differed during the four training conditions. There was indeed a significant main effect of condition (*F*(3,196) = 13.10, *p* < .001, *η* ^2^_p_ = 0.17). Six post‐hoc t‐tests showed that accuracy was significantly lower in the Audio‐Only training condition compared to all three audiovisual training conditions (ts > 3.92, ps < .001, Cohen's *d*s > 0.78). Accuracy for the three audiovisual training conditions was not significantly different (ts < 0.57, ps > .592, Cohen's ds < 0.11; see Figure 2a), although ceiling effects are present that could mask potential effects of gaze. During training, it is therefore perhaps unsurprisingly advantageous for participants to have access to an additional source of identity information from the face compared to only being able to hear only the voice.

### Accuracy during the recognition task at test

To examine whether participants were able to recognize the learned voice identities in each condition with above chance (chance level = 50%), we again ran 4 one‐sample t‐tests on the mean accuracy by participant during the recognition task at test. Accuracy for all four conditions was significantly above chance, with participants being on average correct on 72.1% of trials for the Audio‐Only condition (*t*(49) = 18.68, *p* < .001, Cohen's *d* = 2.64), 68.6% correct for the No Gaze conditions (*t*(49) = 14.37, *p* < .001, Cohen's *d* = 2.03), 67.8% correct for the Averted Gaze conditions (*t*(52) = 14.00, *p* < .001, Cohen's *d* = 1.92), and 65.0% correct for the Direct Gaze condition (*t*(46) = 12.05, *p* < .001, Cohen's *d* = 1.75).

We furthermore analysed the mean accuracy at test for each participant in a between‐subjects one‐way ANOVA. There was a significant main effect of condition (*F*(3,196) = 5.31, *p* = .002, *η* ^2^_p_ = 0.08). Six post‐hoc t‐tests (alpha was Bonferroni–Holm corrected for six comparisons) showed that accuracy was significantly higher (7.8% difference) after Audio‐Only training condition compared to accuracy after the Direct Gaze training (*t*(95) = 4.13, *p* < .001, Cohen's *d* = 0.84), indicating that a Face Overshadowing Effect was present. After correction for multiple comparisons, however, accuracy after Audio‐Only training was only numerically (but not significantly) higher compared to the No Gaze (3.5% difference in accuracy; *t*(98) = 1.97, *p* = .051, Cohen's *d* = 0.39) and Averted Gaze conditions (4.3% difference in accuracy; *t*(101) = 2.47, *p* = .015, Cohen's *d* = 0.49).

Regarding differences in accuracy between the audiovisual training conditions, accuracy for the Direct Gaze condition was numerically lower (3.6%) than for the No Gaze condition, although this difference was not statistically significant after correction for multiple comparisons (*t*(95) = 2.01, *p* = .049, Cohen's *d* = 0.41). Similarly, accuracy was 2.8% lower in the Direct vs Averted Gaze conditions, a difference that was again not statistically significant (*t*(98) = 1.56, *p* = .121, Cohen's *d* = 0.31). Finally, accuracy for No Gaze was 0.8% higher than for Averted Gaze but this difference was not significant (*t*(101) = 0.46, *p* = .645, Cohen's *d* = 0.09; see Figure 2b).

In addition to using pairwise t‐tests to assess whether the predicted pattern of results emerges, we additionally ran two further one‐way ANOVAs, the first including the data for Audio‐only, No Gaze and Direct Gaze, the second including the data for Audio‐only, Averted gaze and Direct Gaze. Within these two ANOVAs we could then test our predictions for a graded effect of visual information being present across the sets of three conditions: Specifically, we tested whether accuracy followed the pattern of Audio‐Only > No Gaze (first ANOVA)/Averted Gaze (second ANOVA) > Direct gaze. The data was split up into these two sets of three conditions as we did not have a specific hypothesis of how No Gaze and Averted Gaze conditions would behave in relation to one another. These linear contrasts showed that accuracies indeed followed the predicted patterns in both cases (linear contrast for Audio‐Only > No Gaze > Direct gaze: *t*(144) = 4.01, *p* < .001; linear contrast Audio‐Only > Averted Gaze > Direct gaze: *t*(147) = 3.99, *p* < .001).

Taken together the test data thus suggest that the Face Overshadowing Effect is to some degree modulated by the presence of a visual stimuli and by eye gaze: The Face Overshadowing Effect is most pronounced for direct gaze, reflected in a highly significant difference in accuracy between the two conditions. The size of the effect in terms of the difference in accuracy is, however, halved, when direct gaze is removed, while a face (with closed or averted eyes) remains present during training.

### Unbiased hit rate (D prime) and response bias (criterion C) during the recognition task at test

To account for and describe any potential response biases that may be present in our accuracy data during the recognition task at test (Stanislaw & Todorov, 1999), we furthermore computed D Prime and Criterion C values for each participant.

We first examined whether D Prime values per condition were significantly different from 0, indicating that participants were able to detect the signal: In this study, a D Prime value above 0 would show that participants were able to accurately perceive the learned voices as “old voices”, while not erroneously labelling previously unheard voices as “old voices”. The higher the D Prime value for a participant, the better their performance. D Primes for all four conditions was significantly above 0, with the mean D Prime for the Audio‐Only condition being 1.37 (*t*(49) = 16.19, *p* < .001, Cohen's *d* = 1.45), D Prime for the No Gaze condition being 1.08 (*t*(49) = 13.52, *p* < .001, Cohen's *d* = 1.03), D Prime for the Averted Gaze condition being 1.03 (*t*(52) = 12.77, *p* < .001, Cohen's *d* = 0.90), and D Prime for the Direct Gaze condition being 0.90 (*t*(46) = 12.05, *p* < .001, Cohen's *d* = 0.78).

As for the analysis of accuracy, we examined whether D Prime differences across conditions with a one‐way between‐subjects ANOVA. There was a main effect of condition (*F*(3,196) = 5.89, *p* = .001, *η* ^2^_p_ = 0.08). Post‐hoc t‐tests (alpha Bonferroni–Holm corrected for six comparisons) showed that D Prime values were significantly higher in the Audio‐Only training condition compared to both the Direct Gaze (*t*(95) = 4.02, *p* < .001, Cohen's *d* = 0.82) and the Averted Gaze conditions (*t*(101) = 2.80, *p* = .006, Cohen's *d* = 0.55). However, while D Prime values were numerically lower for the No Gaze vs the Audio‐Online condition, this difference was not statistically significant after correction for multiple comparisons (*t*(98) = 2.36, *p* = .020, Cohen's *d* = 0.48).

**FIGURE 3:**
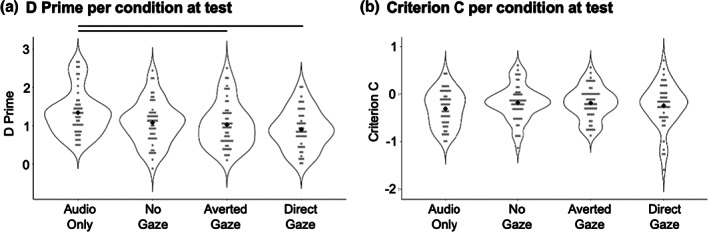
(a) D Prime per participant plotted by condition at test. (b) Criterion C per participant plotted by condition. Grey dots show means for individual participants. Black diamonds show the group means. Lines show significant pairwise comparisons after Bonferroni–Holm correction after six comparisons.

Among the three audiovisual training conditions, a similar picture to the one reported for accuracy above emerges: While D Prime values for the Direct Gaze condition were numerically lower than for the No Gaze condition, this difference was not statistically significant (*t*(95) = 1.64, *p* = .105, Cohen's *d* = 0.33). The same is true for D Prime for Averted Gaze vs Direct Gaze (*t*(98) = 1.15, *p* = .251, Cohen's *d* = 0.23). Finally, D Prime was again marginally higher for the Eyes Closed vs Averted Gaze condition (*t*(101) = 0.46, *p* = .649, Cohen's *d* = 0.09; see Figure 3a).

We also conducted the same linear contrast analysis described for the accuracy data on the D Prime data. These linear contrasts showed that D Prime also followed the predicted patterns for both contrasts (linear contrast for Audio‐Only > No Gaze > Direct gaze: *t*(144) = 4.00, *p* < .001; linear contrast Audio‐Only > Averted Gaze > Direct gaze: *t*(147) = 3.94, *p* < .001).

To describe the nature of any potential response biases, we finally examined whether Criterion C per condition was different from 0: If Criterion C were significantly below 0, this would show that participants were significantly biased towards responding with “old voice”, while C significantly above 0 would indicate a significant bias towards responding with “new voice”.

Criterion C for all three conditions was significantly below 0, with the mean Criterion C for the Audio‐Only condition being −0.43 (*t*(49) = 6.01, *p* < .001, Cohen's *d* = 2.19), C for the No Gaze condition being −0.10 (*t*(49) = 3.52, *p* < .001, Cohen's *d* = 1.85), C for the Averted Gaze condition being −0.19 (*t*(52) = 4.01, *p* < .001, Cohen's *d* = 2.03), and C for the Direct Gaze condition being −0.25 (*t*(46) = 3.62, *p* < .001, Cohen's *d* = 1.60). While Criterion C did not differ by condition as indicated by a one‐way between‐subjects ANOVA (*F*(3,196) = 1.23, *p* = .298, *η* ^2^_p_ = 0.02; Figure 3b), this analysis nonetheless indicates a consistent bias towards perceiving voices as previously learned across all conditions.

## DISCUSSION

In the current study, we investigated whether automatic attention capture by faces can modulate how audiovisual training affects voice identity learning. We find Face Overshadowing Effects in our study, where voice identity recognition after audiovisual training was less accurate than voice identity recognition after audio‐only training. Indeed, this effect was modulated by the direction of eye gaze of a face presented during audiovisual training: The Face Overshadowing Effect was most pronounced when the audiovisual training included faces exhibiting direct gaze, resulting in a significant difference in accuracy between the Direct Gaze and Audio‐Only conditions at test. Conversely, the size of the Face Overshadowing Effect was roughly halved in terms of the difference in accuracy, when no direct gaze was present during training, leading to non‐significant differences between accuracy after Audio‐Only vs No Gaze and Averted Gaze training. When comparing the three different audiovisual conditions, accuracy was numerically lower for faces exhibiting direct gaze compared to faces with no gaze (eyes closed) and averted gaze. These patterns of results are reflected in both the analysis of accuracy data and the analysis of the signal detection measure D Prime for voice identity recognition at test.

In the context of conflicting results in the literature where the presence of a face has been shown to both help and hinder voice identity learning, it is worth first considering how finding a Face Overshadowing Effect in the current study fits within that literature. With there being only limited work studying how the design choices affect the effects of audiovisual training on voice learning, no strong predictions could be made from the existing literature. However, the nature of the faces shown (dynamic vs static) and the duration of exposure have been highlighted as potentially important design choices. Thus, according to the findings of Zäske et al. (2015), Face Overshadowing effects are more likely observed when static images of faces are used and when the training is very brief. Dynamic stimuli and longer training durations (training of ~30 s of exposure), result in Face Benefits. Tomlin et al. (2017), who find a face overshadowing effect after brief exposure, however, report that there is no difference in the size of this effect for static and dynamic stimuli (without manipulating exposure duration). In the context of having used statistic stimuli in the current study, finding Face Overshadowing effects is perhaps the more likely outcome. The effect of exposure duration remains, however, intriguing. In the current study, participants were presented with each of the voices for around 80 s during training, alongside static images. This exposure duration clearly exceeds the longest durations sampled by Zäske et al. (2015) or any other studies reporting Face Overshadowing effects. If there is a linear relationship between exposure durations and the size of Face Overshadowing Effects and Face Benefits, our study should have resulted in a Face Benefit.

Our findings thus highlight that the duration of exposure or type of stimuli, on their own or in conjunction, are unlikely to fully explain the differences in effects observed in the literature. They instead suggest that other and/or additional factors are at work to modulate whether audiovisual training helps or hinders voice identity learning. The nature of these factors is currently unclear and cannot be determined from the data collected for this study. It is however likely that these factors involve complex interactions between the experimental design of training and test tasks (duration of the training, tasks during training, task for the test phase, etc.) as well as the kind of stimuli used in both the auditory and visual domain (static or dynamic faces, relatively easy or harder to learn voice stimuli, etc.).

In this study, we primarily set out to investigate whether a manipulation of the degree of automatic attention capture associated with a face can modulate the effect of audiovisual training on voice identity learning. By manipulating eye gaze, we predicted that an increased attention capture associated with direct eye gaze should enhance any effect of audiovisual training on voice identity learning (Frischen et al., 2007; Mares et al., 2016; Senju & Hasegawa, 2005). Specifically, in the context of the Face Overshadowing Effect observed in the data, we would have thus expected lowest accuracy for the Direct Gaze condition, highest accuracy for the Audio‐Only condition and intermediate accuracy for the No Gaze and Averted Gaze conditions. This pattern of results was indeed observed in our data as predicted. However, the size of these effects was overall small, such that we only observed statistical differences when comparing accuracy after audio‐only training and audiovisual training including images with direct gaze. Removing the direct gaze during training, by introducing averted gaze or images of faces with their eyes shut, resulted in intermediate levels of accuracy, where the differences in accuracy to both the Audio‐Only and Direct Gaze were not significant.

A strict interpretation of these findings, taking only statistical difference into account, would thus lead to the conclusion that Face Overshadowing Effect only consistently exists when direct gaze is present during training. When a face without direct gaze is present during training, no (significant) Face Overshadowing Effect occurs. This interpretation alone would already indirectly support our predictions that the Face Overshadowing Effect may be underpinned by attentional mechanisms. However, while being parsimonious, this interpretation does not account for the fact systematic numerical differences exist between the Audio‐Only vs less attention‐grabbing No Gaze and Averted Gaze conditions and that these differences consistently go in the predicted directions. We therefore argue that the results should be cautiously interpreted in a graded manner, showing that the presence of a face per se (be it with averted gaze or no visible gaze) leads to a small Face Overshadowing Effect, while the addition of direct gaze enhances this effect. Expressed in slightly broader terms, our results indicate that the presence of a face and changes in eye gaze affect the size of the Face Overshadowing Effect. This latter explanation is also supported by the linear contrasts applied to the ANOVAs, showing that accuracy and D Prime significantly follow the predicted pattern of Audio‐Only > No Gaze/Averted Gaze > Direct Gaze.

In light of this, the current results thus provide some support for the proposal put forward in previous work (Cook & Wilding, 1997, 2001; Stevenage et al., 2011; Tomlin et al., 2017) that the Face Overshadowing Effect may be at least partially underpinned by automatic attention capture by the faces during audiovisual training. In the context of this study, the presence of a face per se captured some attention, while the direct gaze (compared to averted gaze and no gaze conditions) might have then automatically drawn additional attention away from the voice and towards the face during training.

However, the effect of eye gaze on the Face Overshadowing Effect is overall small, resulting in a 2.8% difference in accuracy for Direct Gaze vs Averted Gaze and a 3.6% difference in accuracy for Direct Gaze vs No Gaze. While we do see very small differences in accuracy that suggest that the No Gaze condition may indeed be a more salient and suitable ‘control’ condition that is associated with less attention capture than direct gaze or Averted Gaze condition (e.g. Framorando et al., 2016; Riechelmann et al., 2021), it is possible that the eye gaze manipulation in our experiment was not the most salient manipulation to test attention capture. For example, Tomlin et al. (2017) have shown that is possible to entirely abolish the Face Overshadowing Effect by simply inverting the images of the faces presented, thus observing substantial effects by relatively simple changes to the images. When considering possible alternative manipulations of how attention‐grabbing the face images are, manipulations of the emotional content of the faces come to mind. However, when introducing, for example, angry or threatening faces as attention‐grabbing stimuli, the problem arises that this manipulation introduces the confound of an emotion mismatch between the face and the voice. While potentially less salient, direction of gaze can be perceived as largely independent from the tone of the voices and was thus deemed a more suitable type of manipulation. Overall, while some of the inferences drawn in this study partially rest upon non‐significant differences, these current results of course need to be replicated. Such a future replication study would likely require a substantially larger sample to fully power for the small effects observed. Alternatively, a future replication may want to implement a within‐subjects design, as the between‐subjects design may have made it more difficult to detect the effects of eye gaze in the presence of sizeable individual differences in voice identity learning (see Figure 3).

Within the context of the broader literature, it has previously been shown that the Face Overshadowing Effect is face‐specific and is not a more general effect of any kind of visual stimulus capturing attention in the context of a voice‐learning task (Tomlin et al., 2017). Thus, the Face Overshadowing Effect, just like a “Face Benefit” through audiovisual training, can be interpreted as evidence that participants may automatically and by default form rich, multimodal representations of people when encountering new identities (Maguinness et al., 2021; Von Kriegstein et al., 2008). However, contrary to some proposals, having formed such an audiovisual representation of a person is not always advantageous at retrieval. In our and other studies, the Face Overshadowing Effect shows that the additional and highly salient identity‐related information in a face interferes with the encoding of the to‐be‐learned voices. This observation aligns well with findings that visual information usually dominates over auditory information during perception – and, specifically, identity perception from faces and voices (see Barsics, 2014; Young et al., 2020). Thus, any advantages of learning voices in multimodal settings seem to disappear when increasing differences in the salience of the auditory and visual modalities. A candidate mechanism that may underpin or trigger such visual dominance may be automatic attention capture. Visual dominance and associated attention capture can thus at least in part explain the Face Overshadowing Effect reported in the literature. Conversely, it can also explain why there is no “Voice Overshadowing Effect”, where accuracy for face recognition is similar to when faces have been learned on their own or in the presence of voices (Stevenage et al., 2011): In this case, the identity‐related information from the face is sufficient, such that the relatively less salient and informative information from the voice does not capture attention and is not enhancing face identity learning (see also Lavan, Collins, & Miah, 2021 for audiovisual identity perception).

In the context of a relatively small literature and conflicting findings around the effect of audiovisual training on voice identity learning, more work is needed to build up a more complete picture of how voices and faces interact during identity learning ‐ and identity perception more generally. Similarly, while our study provides some evidence that automatic attention capture may indeed underpin at least modulate the Face Overshadowing Effect, more work aimed at describing the mechanisms supporting the interaction of auditory and visual modalities during identity perception is required.

## AUTHOR CONTRIBUTIONS

**Nisha Ramanik Bamaniya:** Conceptualization; formal analysis; writing – original draft; writing – review and editing. **Raffaella Lucy Monica Price:** Conceptualization; formal analysis; writing – original draft; writing – review and editing. **Moha‐Maryam Muse:** Conceptualization; formal analysis; writing – original draft; writing – review and editing. **Isabelle Mareschal:** Visualization; writing – original draft; writing – review and editing.

## CONFLICT OF INTEREST

All authors declare no conflict of interest.

## Data Availability

Data are available from the corresponding author upon request.
